# Use of cariprazine as an impulsivity regulator in an adolescent with non suicidal self-injury and suicidal attempts. Case report

**DOI:** 10.1192/j.eurpsy.2023.1553

**Published:** 2023-07-19

**Authors:** P. Del Sol Calderon, A. Izquierdo de la Puente, R. Fernández Fernández, M. García Moreno

**Affiliations:** 1 Psychiatry, Hospital Puerta de Hierro, Majadahonda; 2 Psychiatry, Hospital Infanta Cristina, Madrid, Spain

## Abstract

**Introduction:**

Adolescents with emotional dysregulation are at risk for self-injury. Antipsychotics are often used to manage these symptoms.

**Objectives:**

The aim of the present case is to show the use of cariprazine as an effective drug for emotional dysregulation and impulsivity in a 17-year-old adolescent girl

**Methods:**

Case report

**Results:**

The patient was a 17-year-old female admitted to in patient psychiatric unit for a self-harm attempt due to sertraline overdose. She was being followed up for self-injury, anxiety and eating disorder symptoms. Her treatment was sertraline 200 mg, diazepam 20 mg per day and olanzapine 15 mg per day. With this medication she had gained up to 7 kgs in 4 months. A progressive change was made with cariprazine up to 3 mg and olanzapine was reduced to 2.5 mg at night. With this adjustment the patient did not present worsening in anxiety levels, with adequate impulse control and being able to perform emotional regulation strategies.

**Conclusions:**

Although it has no indication in patients under 18 years of age, it shows a case of good tolerance and efficacy for the management of impulsivity by improving emotional regulation. Cariprazine is an atypical antipsychotic that works through partial agonism on dopaminergic receptors, serotonin 5-HT_1A_ receptors and an antagonist at the 5-HT_2B_ receptors, with moderate affinity for adrenergic, histaminergic, and cholinergic receptors reducing the likelihood of side effects

**Disclosure of Interest:**

None Declared

